# Characterizing Core Journals in Ophthalmology Literature Using Bradford’s Law: A Bibliometric Analysis^☆^

**DOI:** 10.62199/2475-4757.1038

**Published:** 2025-03-24

**Authors:** Leo Meller, Vasan Jagadeesh, Michael Oca, Katherine Wilson, Jeffrey Lee, Sally Baxter, Nathan Scott

**Affiliations:** a School of Medicine, University of California San Diego, La Jolla, CA, USA; b Viterbi Family Department of Ophthalmology and Shiley Eye Institute, University of California San Diego, La Jolla, CA, USA; c Division of Biomedical Informatics, Department of Medicine, University of California San Diego, La Jolla, CA, USA

**Keywords:** Medical education, Bibliometric analysis, Ophthalmology, Core journals, Bradford’s law

## Abstract

**Purpose:**

The exponential growth of ophthalmology research can be challenging to keep up with for trainees, researchers and clinicians and little has been done to identify core ophthalmology journals. We thus applied Bradford’s law to characterize core journals in ophthalmology.

**Design:**

Cross-sectional bibliometric analysis.

**Methods:**

Two sets of top 10 National Library of Medicine indexed ophthalmology journals based on h-index and impact factor (IF) were combined to create top 14 journals used for analysis. The references from all articles published in these journals in one randomized quarter were compiled into a citation database. Bradford’s law was applied to identify zonal distribution of journals.

**Results:**

A total of 3093 journals containing 34,633 articles were cited in ophthalmology literature from July–Sept 2022. Under linear regression, IF was significantly associated with h-index for the top 20 journals (P < 0.001) and top 10 ophthalmology journals (P = 0.017). H-index was significantly associated with number of citations for top 10 ophthalmology journals (P < 0.001). Three zones obeying Bradford’s law were identified, with zone 1 (core) containing six journals and 8410 citations, zone 2 containing 27 journals and 8359 citations, zone 3 containing 154 journals and 8419 citations. A linear relationship between the log journal rank for zones 1–3 and cumulative number of citations was found (R^2^ = 0.996), validating Bradford’s law.

**Conclusions:**

Six core ophthalmology journals were identified: *American Journal of Ophthalmology, Investigative Ophthalmology* & *Visual Science, Ophthalmology, JAMA Ophthalmology, British Journal of Ophthalmology, Journal of Cataract* & *Refractive Surgery*. These highly cited core journals may serve as high-yield resources for describing the latest advances in ophthalmology research and may guide ophthalmology trainees and educators with their learning and teaching.

## Introduction

Clinicians and educators frequently use evidence found in peer-reviewed publications to effectively address the health problems caused by diseases within their patient populations.^1–3^ In our modern era of global interconnectivity, ophthalmic research is driven by contributions from individuals and institutions worldwide. Yet, with over 10,000 ophthalmology articles per year in North America alone, specialists face mounting difficulties in keeping up with the expanding volume of knowledge in their field.^4^ This also poses a challenge for ophthalmology trainees as well, who may be overwhelmed by the large volume of ophthalmology publications and face an increasing difficulty in selecting the appropriate journals to read as a part of their training, which is critical for them to learn the skills of evidence-based medicine.^5,6^

Common metrics that are widely accepted to compare the quality of information in academic journals are the impact factor (IF) and the h-index.^7,8^ IF reports the average number of citations each article within a given journal received in the past two calendar years.^9^ The h-index provides the highest value in which the number of published articles is equal to or greater than the number of citations.^10^ However, IF and h-index do not help identify a core set of journals with the highest yield information that is relevant for the clinical practice and education of ophthalmology.^8,11^ Currently, little has been done to explore the essential journals that eye and vision trainees, researchers and clinicians should follow, and an evidence-based recommendation on this topic is crucial for trainees and clinicians to stay updated on the latest findings in their respective fields.^12–14^ A potential solution is to utilize bibliometric analysis, a quantitative method that can be used to evaluate the research outputs of various journals, such as in the field of eye and vision research.^15^ By analyzing elements such as the source, format, type, and citation count of published journal articles, we can gain valuable insights into the scholarly impact of these journals. This allows us to determine which specific research studies and journals have had a notable impact and garnered attention within the field of eye and vision research.^16,17^

Presently, we aim to address this knowledge gap and characterize the journals of highest utility in ophthalmology using Bradford’s law of scattering. Bradford’s law, conceptualized by Samuel C. Bradford in 1934, states that the number of articles on a subject follows a logarithmic distribution and the journals in which they are from can be divided across different zones based on citation patterns, with a core set (first zone) of journals publishing the majority of relevant articles.^18^ In this context, relevance is defined as how frequent articles from a given journal are cited by other articles, with journals that receive more citations considered more valuable. The law illustrates the concept of diminishing returns, as the most relevant set of core journals (zone one/core zone) contains the least number of journals yet most heavily cited (i.e. these journals publish articles that are more frequently cited), whereas the least relevant zone three contains the greatest number of journals yet are least cited (i.e. these journals publish articles that are less frequently cited). Thus, as one moves from the core journals (zone one) to zone three, one will find more and more journals, but fewer articles in these journals are cited. Bradford’s law is thus able to generate a discipline-specific list of core journals, which effectively improves upon the ranking established by IF and h-index, which are more general and fail to consider the specific citation pattern within a given discipline. Therefore, our study applied Bradford’s law to ophthalmology literature to identify core journals in ophthalmology, which may aid trainees and eye and vision researchers in keeping up with the latest advancements in ophthalmology.

## Methods

In this cross-sectional bibliometric analysis, we followed the methodology established by Madhugiri et al. in their characterization of neurosurgery core journals using Bradford’s law.^19^ The two main steps, detailed below, include generation of a citation database and application of Bradford’s law to identify core journals. We performed linear regression to assess the associations among citation database outcomes, h-index, and IF. Microsoft^®^ Excel (Version 16.64) was used to perform all analyses. Institutional Review Board review and approval was not required for this study as no patient or participant information was utilized and all analyzed data are publicly available. We confirm that this study adheres to the Declaration of Helsinki and all federal or state laws in the United States of America.

### Citation database generation

The first step is to establish a citation database containing all the “useful” articles in the present ophthalmology literature. Set forth by Madhugiri et al.,^19^ “useful” articles are operationalized as the articles that are cited (included in the reference list) by other articles in the same discipline, which reflects their contribution to the advancement of clinical care and research in this very discipline. Therefore, this citation database enables us to identify which journals are supplying the most “useful” articles and allows us to identify the core journals in this discipline.

We first identified all MEDLINE indexed ophthalmology journals in the National Library of Medicine catalog. Next, we identified the IF (Clarivate Analytics 2022) and h-index (Scopus 2022, Elsevier B.V) for each indexed ophthalmology journals. We then ranked all of these journals based on IF and h-index, to which two top 10 rank lists were established, one for IF and the other for h-index. To ensure a broad representation of ophthalmologic literature and to reduce bias towards any given subspecialty within ophthalmology (i.e. neuro-ophthalmology, pediatric ophthalmology), we followed the methodology described in Madhugiri et al. and only the highest ranked journal in each subspecialty (in this case, cataract and refractive surgery and Neuro-Ophthalmology) was included for each rank list.^19^ We also excluded optical journals, such as *Optics Express,* to ensure that we focused on medical ophthalmology journals that are most utilized by general clinicians and medical researchers. These two rank lists were then combined by including journals at the intersection of both rank lists and adding new journals that were not included by each individual rank list, and we again excluded optical journals and over-representation of subspecialties. This led to 14 final journals utilized to generate the citation database.

We then listed all quarters in the past three calendar years at the time of the analysis (Jan 2023), which included January 2020–December 2022. Again, to ensure a more comprehensive view of ophthalmology literature and to prevent over-representation of any discussions, topics or subspecialties, we excluded any quarter in which one of the 14 journals only published a specialty or supplement issue. Following the method established by Madhugiri et al.,^19^ to improve feasibility of our study, we then selected one randomized quarter of the remaining quarters, which was July–Sept 2022. We proceeded to compile the articles and their characteristics (i.e. in which journal they were published) referenced in all published articles (excluding editorials and letters to the editor) in all 14 journals during July–Sept 2022, generating the citation database.

### Bradford’s law application and core ophthalmology journal identification

Within the citation database, we ranked all included journals based on the number of articles supplied by each journal (the highest ranked journal provided the greatest number of references). We then applied Bradford’s law to identify a zonal distribution of all journals in the citation database obeying the distribution of c:ck:ck,^2^ where c represents the number of journals in the core zone and k is an integer multiplier. As the number of “useful” articles within each zone (c or ck or ck^2^) is expected to be similar, this zonal distribution based on Bradford’s law illustrates the concept of diminishing return, as when one navigates away from the core zone (c), a significantly higher number of journals based on k is needed to supply a similar number of useful articles. Following Madhugiri et al.,^19^ we systematically tested different values for c and k starting with c = 3 to establish the pattern of c:ck:ck^2^ within our citation database, and were able to identify core journals in the ophthalmology literature in a distribution manner that obeys Bradford’s law.

### Self-referencing bias detection and result validation

Consistent with previous applications of Bradford’s law,^19,20^ we performed self-citation analysis using a cutoff of 20 % for self-citation bias for the initial 14 journals utilized to compile the citation database to detect any bias in self-referencing,^21^ and log scale analysis to validate our zonal distributions identified by Bradford’s law.

## Results

### Citation database generation

**Fig. 1.**
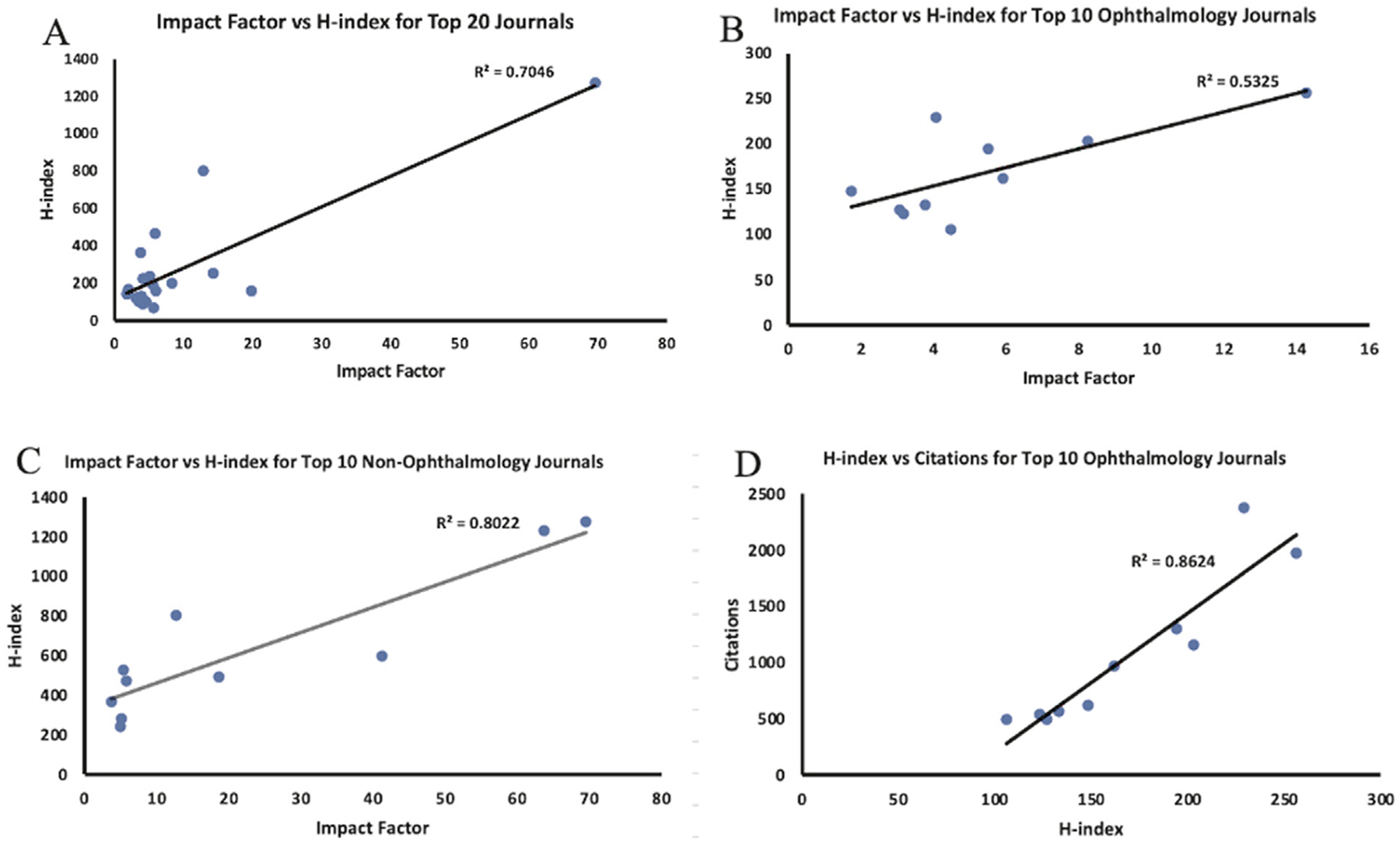
Correlation between impact factor, h-index, and citations. A–C: Impact factor is significantly correlated with h-index for top 20 journals in citation data base (P < 0.001), for the top 10 ophthalmology journals (P = 0.017), for the top 10 non-ophthalmology journals (P < 0.001). D: H-index is significantly correlated with number of citations in citation data base for top 10 ophthalmology journals (P < 0.001).

After combining the h-index and IF rank list as detailed above, 14 journals were utilized to generate the citation database (Table 1) and no self-referencing bias exists for these 14 journals (Table 2). A total of 34,633 articles from 3093 journals were cited in the citation database between July to September 2022. Under linear regression analysis, based on ranking established within the citation database based on number of articles supplied by each journal, IF was significantly correlated with h-index for the top 20 journals overall (P < 0.001) and top 10 ophthalmology journals (P = 0.017), and for the top 10 non-ophthalmology journals (P < 0.001) in the citation database (Fig. 1A–C). H-index was significantly correlated with the number of citations in the citation database for the top 10 ophthalmology journals (P < 0.001) (Fig. 1D).

### Bradford’s law application and core ophthalmology journal identification

**Fig. 2.**
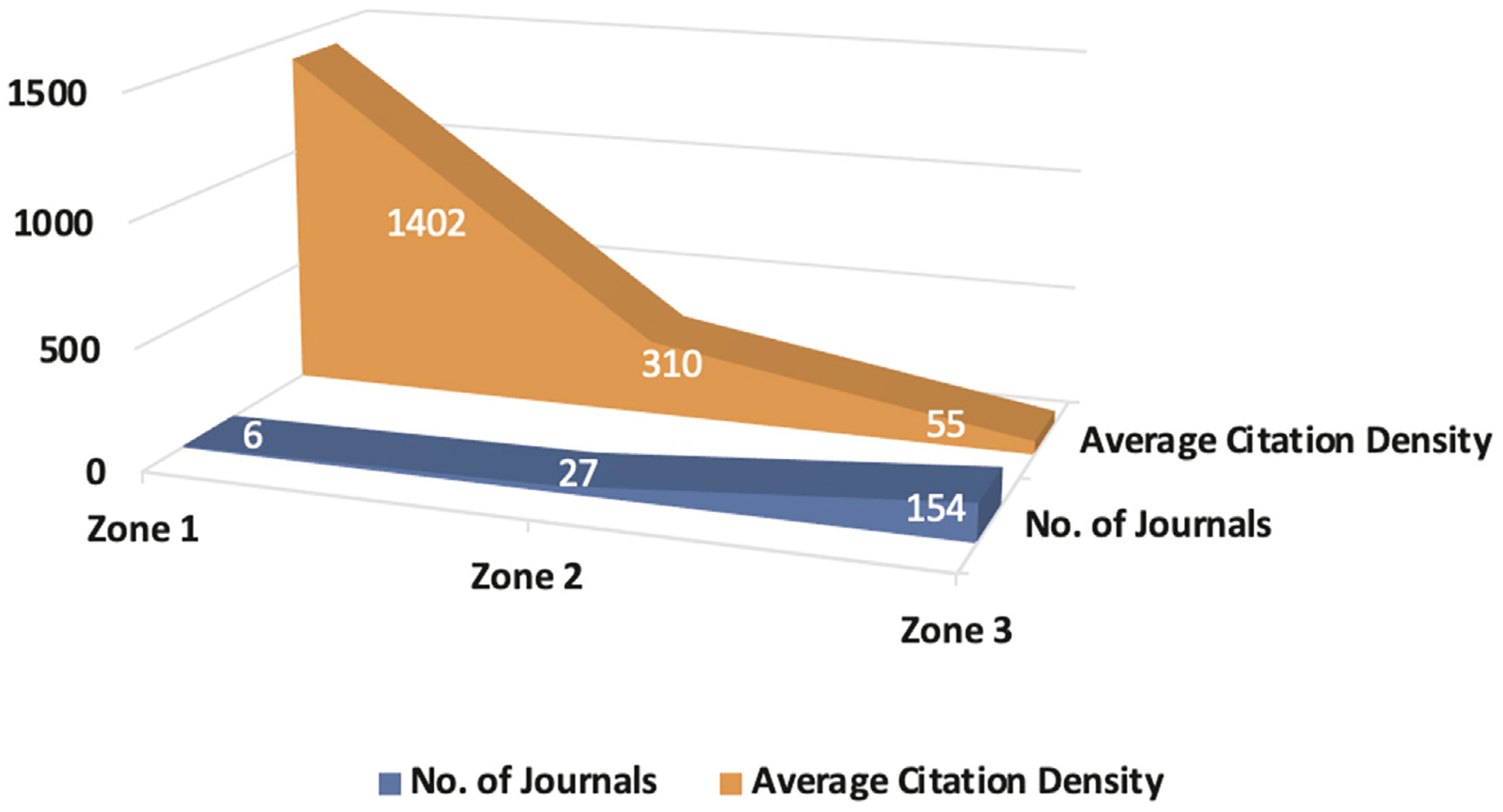
Graphical illustration of Bradford’s law zonal distribution. The graph illustrates the concept of diminishing returns, as the most relevant set of core journals (zone 1) contains the least number of journals yet has the highest average citation density, whereas the least relevant zone 3 contains the most number of journals yet the lowest average citation density. Citation density is defined as average number of citations in a given zone.

**Fig. 3.**
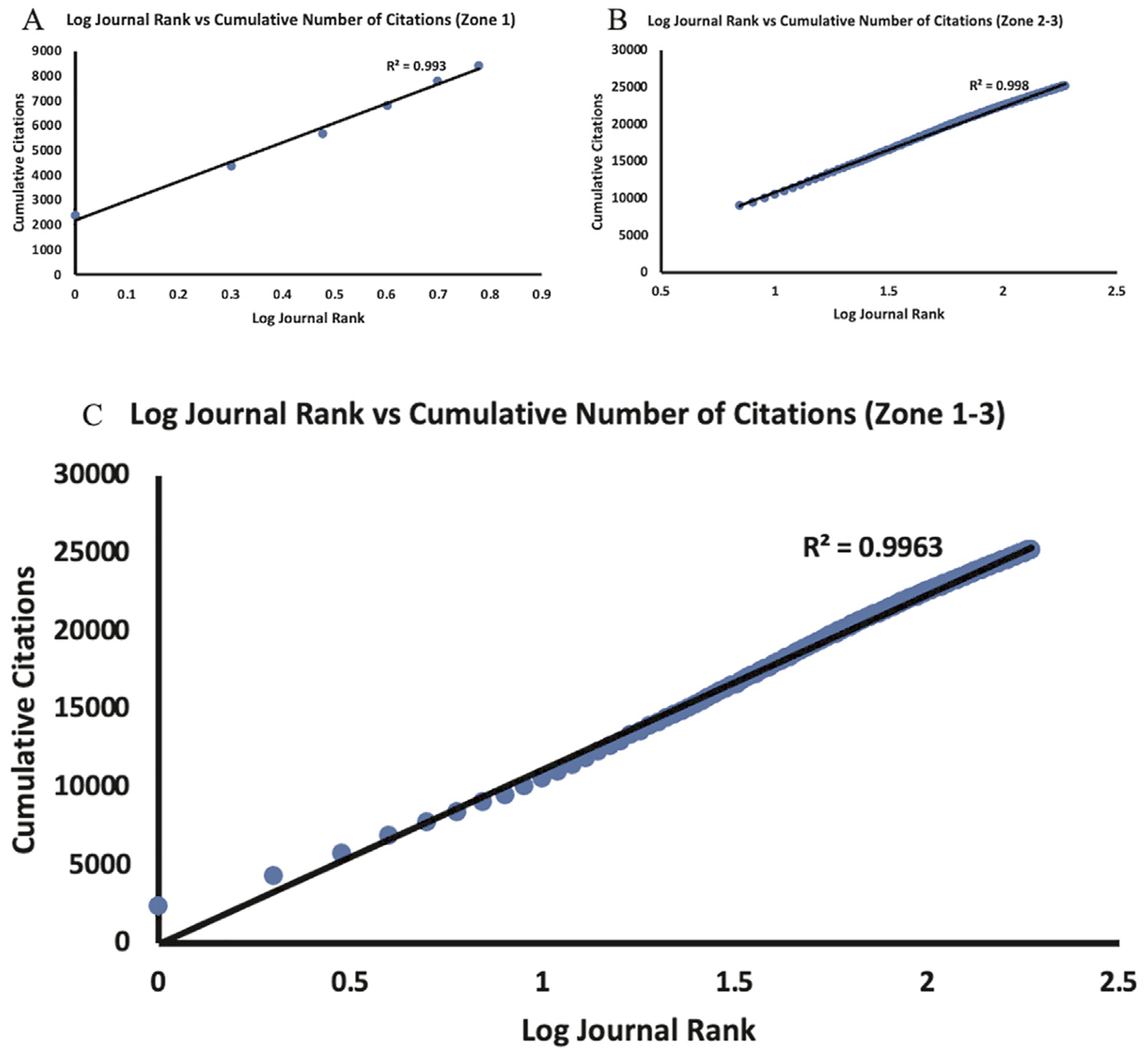
Log journal rank versus cumulative number of citations. A–C: Linear relationship between log journal rank and cumulative number of citations was observed for Zone 1–3, Zone 1 and Zone 2–3.

As described above, we applied Bradford’s law to identify a zonal distribution that observes c:ck:ck^2^ by testing different values of c and k. Following Madhugiri et al.,^19^ we first tested c = 3, representing three core journals that yielded 5633 citations. We then identified the second and third set of journals that yielded the closest number of citations to 5633, which respectively were 9 journals yielding 5740 citations (zone 2) and 23 journals yielding 5650 citations (zone 3). However, this distribution of 3:9:23 does not obey c:ck:ck^2^ for Bradford’s law, as we were unable to identify a suitable integer multiplier k. Hence, c = 3 was discarded. As the top 10 journals in the citation database were all ophthalmology journals, we then tested different values of c from 4 to 10. Only when c = 6 were we successful in identifying a zonal distribution that obeys c:ck:ck^2^ for Bradford’s law. The top six journals (core journals) yielded 8410 citations for zone 1, 27 journals in zone 2 yielded 8359 citations and 154 journals in zone 3 yielded 8419 citations (Supplementary Table 1). Hence, we identified six core ophthalmology journals based on Bradford’s law, which include: *American Journal of Ophthalmology, Investigative Ophthalmology* & *Visual Science, Ophthalmology, JAMA Ophthalmology, British Journal of Ophthalmology, Journal of Cataract* & *Refractive Surgery* (Table 3). We provide a visual illustration of this zonal distribution demonstrating Bradford’s law (Fig. 2) and verified that this zonal distribution obeyed Bradford’s law through log-scale analysis, as linear relationships between log journal rank and cumulative number of citations exist for proposed zones 1–3, zone 1, and zones 2–3 (Fig. 3).

## Discussion

### Core journals

To provide optimal care, ophthalmologists need to stay up to date with medical literature.^22,23^ Since their inception, medical journals have represented the most rapidly expansive field of journal publications globally.^23^ With one newly published article every 26 s, it would be nearly impossible for ophthalmologists to follow all relevant literature.^23^ Therefore, identifying key ophthalmology journals are critical. Previous studies focused on most frequently cited articles in ophthalmology,^24^ but little has been done to identify core ophthalmology journals that researchers and clinicians should prioritize. Bradford’s law, used across fields such as physical therapy,^25^ knowledge economy,^26^ pediatric neurosurgery,^27^ neurosurgery,^19^ and otolaryngology,^20^ provides a framework for identifying such core journals. We applied Bradford’s law to investigate the core journals in the realm of ophthalmology literature.

We uncovered the six core journals in ophthalmology literature: *American Journal of Ophthalmology, Investigative Ophthalmology* & *Visual Science, Ophthalmology, JAMA Ophthalmology, British Journal of Ophthalmology, Journal of Cataract* & *Refractive Surgery*. All journals target a readership of ophthalmologists and visual scientists. Additionally, aside from *Journal of Cataract* & *Refractive Surgery*, which uniquely discusses anterior segment surgery, the remaining journals demonstrated a comprehensive scope. The fact that five of six core journals disseminate general ophthalmic knowledge demonstrates that Bradford’s law can identify journals that will provide clinicians with the most comprehensive picture of the current field of ophthalmology. This notion is further supported by previous studies on Bradford’s law,^19,20^ which also primarily identified comprehensive core journals in their respective fields. Nonetheless, the inclusion of specialized journals in the core list is also important. Although the scope of *Journal of Cataract* & *Refractive Surgery* is highly specific, cataracts are the leading cause of blindness worldwide and the leading cause of vision loss in the United States.^28^ In fact, cataract surgery is the most commonly performed surgery in the United States.^29^ In addition, refractive errors are the most frequent cause of eye disorder in the United States.^28^ Hence, the topics discussed in this journal may seem discrete but in fact represent the most common eye disorders within the field of ophthalmology.

### Power analysis, methods and results validation

For practicality and to reduce sampling bias, we randomly selected one quarter for analysis. Of all prior research using Bradford’s law, our randomly selected sample included the greatest number of articles (34,633 compared with 5467,^30^ 8411,^31^ 11,319,^32^ 16,518,^25^ 22,850,^19^ and 26,876 articles^20^), and thus demonstrated the highest power and the increased robustness of the present results.

In regard to self-referencing bias, only one journal from our list of the top 14 journals, *Journal of Cataract* & *Refractive Surgery*, had a self-citation percentage greater than 20 %. This is not unusual based on previous reports in other specialties,^19,20^ and when the specificity of *Journal of Cataract* & *Refractive Surgery* is considered. The limited number of journals on subspecialty topics, in this case the subject of cataracts and refractive surgery, may justify its higher self-citation rate; however, a self-citation percentage of greater than 20 % in a journal with a broader scope (i.e., *Ophthalmology*) may indicate bias.

Log scale analysis validated our zonal distributions identified by Bradford’s law. It has been demonstrated by Madhugiri et al.^19^ that a linear association between log journal rank and cumulative number of citations indicates obedience to Bradford’s law: to maintain the same number of relevant citations while moving away from the core zone of journals, an exponential increase in the number of journals is needed. This log scale analysis therefore validates our identification and proposal of three zonal distributions within ophthalmology literature (Fig. 3). Therefore, our methodology and analysis remain robust after consideration of sample size and power, self-citation bias, and log-scale verification of Bradford’s law.

### Correlation between h-index, impact factor, and citation density

There was a significant correlation between h-index and IF within the top 20 journals of our citation database (Fig. 1A). The data visualized in Fig. 1B and C provide further supportive evidence for the significant correlation between h-index and IF, demonstrating a positive correlation between h-index and IF for the top 10 ophthalmology journals and the top 10 non-ophthalmology journals we evaluated. It is beyond the scope of the present analysis to assess the mechanistic reason behind the correlation between h-index and IF, which may prove discipline specific and should serve as a subject of future research. Fig. 1D showcases the significant correlation between h-index and citation count for the top 10 ophthalmology journals. This association supports the use of Bradford’s law – a method of classifying journals into zones based on citation counts.

### Utility of Bradford’s law in identifying core journals

Conventionally, both IF and h-index have been used as proxies for the quality of a journal. However, this trend of equating high IF and h-index with the quality of scientific works is declining.^33–36^ Furthermore, although both IF and h-index demonstrate the bibliometric patterns of a journal, they do so broadly, and do not consider the use of a given journal amongst any specific discipline. For instance, prestigious journals such as *Nature* and *Science* are highly impactful based on IF and h-index, however, this does not warrant them to be incorporated into the reading list of busy ophthalmologists because the metrics of IF and h-index are established based on general citation patterns by researchers from various disciplines. That is, IF and h-index do not give insight into how useful a journal is to those working in a particular specialty. On the other hand, our application of Bradford’s law to determine the list of core journals in ophthalmology is based upon the citation pattern specific to eye and vision research as our citation database contained only articles cited by ophthalmology literature during the study period, and thus proves discipline specific compared with IF and h-index. Madhugiri et al.^19^ emphasize this point in their application of Bradford’s law to identify core journals in neurosurgery, and stated that, “This analysis could be carried out for every specialty so as to generate a core set of journals for the medical sciences in general.”

As discussed previously,^19^ the primary usefulness of Bradford’s law in identifying these core ophthalmology journals is two-fold. Firstly, the citation database only included ophthalmology articles and therefore is ophthalmology specific. Secondly, Bradford’s law zonally distributes ophthalmology journals, and it is clear how a handful of journals are responsible for the majority of citations in ophthalmology and thus of the highest utility (Fig. 2). Our findings should not be taken to discredit the utility of other ophthalmology journals. Instead, the results emphasize the disproportionate impact of the six core journals we identified herein.

### Relevance to ophthalmology medical education

Reading and synthesizing medical literature is a critical component of medical education and cultivates intellectual inquiry.^37^ For educators, it is also necessary to integrate evidence-based medicine as a part of their teaching^38^ and to ensure new discoveries are disseminated in a timely manner. In a survey study, Algra and Dekker^39^ identified that medical students begin to read scientific literature early on in their curriculum, particularly during clinical rotations or when they begin research, and 60 % of students on clinical rotations read at least one journal a few times each month. However, the rapid establishment of new ophthalmology journals and publication of articles can pose a challenge for trainees to decide what to read. Notably, in a multi-institutional study by Kind et al.,^40^ the authors reported that 11 % of students did not know what to read on clerkships and that students struggled to identify proper resources. Therefore, from the perspective of ophthalmology education, it is especially critical to identify high-yield resources for trainees.

The 6 core journals identified herein were the most densely cited among ophthalmology literature. As discussed previously, 5 of 6 journals are comprehensive and thus will enable trainees to gain a broad understanding of the latest advancements in the field. Moreover, the inclusion of *Journal of Cataract* & *Refractive Surgery*, although highly specific, enables trainees to remain up to date with advancements in some of the most common procedures they will observe and perform. For comprehensive education, we recommend trainees to incorporate these core journals as a part of their learning and for educators to emphasize the latest reports in these core journals. Given the lack of other subspecialty journal representation in this core list, we also recommend trainees remain up to date on subspecialty journals (such as *Ophthalmic Plastic and Reconstructive Surgery*) for their relevant clinical encounters and interests.

### Limitations

Our initial approach aimed to reduce subspecialty bias but in doing so, we were not able to perform a comprehensive exploration of the subspecialties. For instance, we excluded optics-based journals, and could not account for emerging topics such as artificial intelligence and Optical Coherence Tomography Angiography.^41,42^ Future research must focus on these specific fields. Furthermore, the scope of our conclusions was restrained by data from one randomized quarter. The landscape of ophthalmology literature may prove ever changing and COVID-19 may have influenced the scope and type of articles that were published during our randomized study time period. However, our sample size of 34,633 is still the largest to date compared to other applications of Bradford’s law, demonstrating the robustness and reliability of our results. Future studies on this topic should focus on more longitudinal data and validate our findings on a more extensive time scale.

In conclusion, research to identify core ophthalmology journals most useful to ophthalmologists and related researchers has been limited. We utilized Bradford’s law to identify six core journals in ophthalmology literature, including *American Journal of Ophthalmology, Investigative Ophthalmology* & *Visual Science, Ophthalmology, JAMA Ophthalmology, British Journal of Ophthalmology, Journal of Cataract* & *Refractive Surgery*. Our analysis was validated after power analysis, self-citation analysis, and log-scale analysis showing obedience to Bradford’s law, and we highlight the utility of these six journals and suggest their incorporation into the reading list of eye and vision researchers and clinicians.

## Supplementary Material

1

## Figures and Tables

**Table 1.** Top 14 journals used for analysis based on impact factor and h-index.

Journal	Citation List Rank	H Index Rank	Impact Factor Rank	Issues in Quarter 3, 2022	H Index	Impact Factor	Citations from Journal	Mean Citations Per Month
*Investigative Ophthalmology & Visual Science*	1	2	11	3	229	4.05	2380	793
*Ophthalmology*	2	1	2	3	256	14.28	1979	659
*American Journal of Ophthalmology*	3	4	8	3	194	5.49	1304	434
*JAMA Ophthalmology*	4	3	3	3	203	8.25	1158	386
*British Journal of Ophthalmology*	5	7	6	3	162	5.91	970	323
*Journal of Cataract & Refractive Surgery*	6	8	14	3	148	1.72	619	206
*Experimental Eye Research*	7	10	12	3	133	3.77	571	190
*Eye - Nature*	8	11	9	3	106	4.46	493	164
*Progress in Retinal and Eye Research*	9	6	1	2	164	19.70	434	217
*Vision Research*	10	5	13	3	171	1.98	334	111
*The Ocular Surface*	11	12	7	1	71	5.57	282	282
*Survey of Ophthalmology*	12	9	5	1	137	6.20	244	244
*Journal of Neuro-Ophthalmology*	13	13	10	1	60	4.42	91	91
*Annual Review of Vision Science*	14	14	4	1	30	7.73	40	40

**Table 2.** Method validation: self-citation analysis.

Journal	Citations retrieved from journal	Self-citations	Self-citation (%)
*Progress in Retinal and Eye Research*	6053	168	2.78
*Experimental Eye Research*	4317	225	5.21
*Survey of Ophthalmology*	4065	52	1.28
*American Journal of Ophthalmology*	3525	280	7.94
*Investigative Ophthalmology & Visual Science*	3146	370	11.76
*Ophthalmology*	2805	428	15.25
*British Journal of Ophthalmology*	2215	153	6.90
*Annual Review of Vision Science*	2070	19	0.92
*Eye - Nature*	1782	111	6.23
*The Ocular Surface*	1285	113	8.79
*Vision Research*	1131	134	11.85
*Journal of Cataract & Refractive Surgery*	1023	275	26.88
*JAMA Ophthalmology*	930	67	7.20
*Journal of Neuro-Ophthalmology*	317	21	6.62

**Table 3.** Core journals in ophthalmology according to Bradford’s law.

Rank	Journal	No. of Citations (Total: 8410)	% of Total Citations (Accumulative: 24.28)	Log-Rank
1	*Investigative Ophthalmology & Visual Science*	2380	6.87	0.00
2	*Ophthalmology*	1979	5.71	0.30
3	*American Journal of Ophthalmology*	1304	3.77	0.48
4	*JAMA Ophthalmology*	1158	3.34	0.60
5	*British Journal of Ophthalmology*	970	2.80	0.70
6	*Journal of Cataract & Refractive Surgery*	619	1.79	0.78
