# Supplementary material for: Characterizing Core Journals in Ophthalmology Literature Using Bradford’s Law: A Bibliometric Analysis
Source: J Acad Ophthalmol (2017). Author manuscript; Available in PMC 2026 Aug 15. (PMC13475947; doi:10.62199/2475-4757.1038)
Supplement: Supplementary file 1 [file NIHMS2202495-supplement-1.pdf]

Supplementary

Supplementary Table 1. Distribution of ophthalmology journals according to Bradford's law.

Rank	Journal	Number of Citations	% of Total Citations	Log-Rank
Zone 1 (Core Zone)				
1	*Investigative Ophthalmology & Visual Science*	2380	6.87	0.00
2	*Ophthalmology*	1979	5.71	0.30
3	*American Journal of Ophthalmology*	1304	3.77	0.48
4	*JAMA Ophthalmology*	1158	3.34	0.60
5	*British Journal of Ophthalmology*	970	2.80	0.70
6	*Journal of Cataract & Refractive Surgery*	619 (8410*)	1.79 (24.28*)	0.78
Zone 2				
7	*Experimental Eye Research*	571	1.65	0.85
8	*Cornea*	542	1.56	0.90
9	*Eye - Nature*	493	1.42	0.95
10	*Retina — The Journal of Retinal and Vitreous Diseases*	493	1.42	1.00
11	*PLOS One*	492	1.42	1.04
12	*Progress in Retinal and Eye Research*	434	1.25	1.08
13	*Proceedings of the National Academy of Sciences (PNAS)*	420	1.21	1.11
14	*Scientific Reports - Nature*	397	1.15	1.15
15	*Acta Ophthalmologica*	354	1.02	1.18
16	*Graefe's Archive for Clinical and Experimental Ophthalmology*	348	1.00	1.20
17	*Vision Research*	334	0.96	1.23
18	*The Ocular Surface*	282	0.81	1.26
19	*Journal of Neuroscience*	269	0.78	1.28
20	*Nature*	267	0.77	1.30
21	*Journal of Biological Chemistry*	252	0.73	1.32
22	*Survey of Ophthalmology*	244	0.70	1.34
23	*Molecular Vision*	229	0.66	1.36
24	*Journal of Glaucoma*	224	0.65	1.38
25	*Clinical Ophthalmology*	223	0.64	1.40
26	*Optometry Vision Science*	217	0.63	1.41
27	*Science/AAAS*	200	0.58	1.43
28	*Current Eye Research*	193	0.56	1.45
29	*Neuron*	190	0.55	1.46
30	*Translational Vision Science & Technology*	189	0.55	1.48
31	*Journal of Refractive Surgery*	174	0.50	1.49
32	*Nature Genetics*	166	0.48	1.51
33	*Human Molecular Genetics*	162 (16769[a])	0.47 (48.42[a])	1.52
Zone 3				
34	*New England Journal of Medicine*	162	0.47	1.53
35	*Clinical and Experimental Ophthalmology*	154	0.44	1.54
36	*JAAPOS*	154	0.44	1.56
37	*The Lancet*	152	0.44	1.57
38	*European Journal of Ophthalmology*	147	0.42	1.58
39	*International Journal of Molecular Sciences*	147	0.42	1.59
40	*Johnson & Johnson Vision*	143	0.41	1.60
41	*Nature Communications*	138	0.40	1.61
42	*BMC Ophthalmology*	130	0.38	1.62
43	*Cell*	124	0.36	1.63
44	*OSLI Retina*	120	0.35	1.64
45	*Current Opinion in Ophthalmology*	119	0.34	1.64
46	*Indian Journal of Ophthalmology*	119	0.34	1.66
47	*JAMA*	119	0.34	1.67
48	*Ophthalmic and Physiological Optics*	119	0.34	1.68
49	*Journal of Ophthalmology*	116	0.33	1.69
50	*Ophthalmology Retina*	114	0.33	1.70
51	*The Journal of Physiology - London*	113	0.33	1.71
52	*Ocular Immunology and Inflammation*	109	0.31	1.72
53	*Nature Neuroscience*	104	0.30	1.72
54	*The American Journal of Human Genetics*	99	0.29	1.73
55	*International Ophthalmology*	96	0.28	1.74
56	*Journal of the Optical Society of America*	95	0.27	1.75

(*continued on next page*)

Supplementary Table 1. (continued)

Rank	Journal	Number of Citations	% of Total Citations	Log-Rank
57	*Diabetes Care*	91	0.26	1.76
58	*Journal of Neuro-Ophthalmology*	91	0.26	1.76
59	*Ophthalmologica*	89	0.26	1.77
60	*Current Biology*	88	0.25	1.78
61	*Diabetes- American Diabetes Association*	86	0.25	1.79
62	*Eye & Contact Lens*	86	0.25	1.79
63	*Transactions of the American Ophthalmological Society*	86	0.25	1.80
64	*Journal of Comparative Neurology*	81	0.23	1.81
65	*Canadian Journal of Ophthalmology*	77	0.22	1.81
66	*International Journal of Ophthalmology*	77	0.22	1.82
67	*Japanese Journal of Ophthalmology*	77	0.22	1.83
68	*Advances in Experimental Medicine & Biology*	72	0.21	1.83
69	*The Journal of Clinical Investigation*	71	0.21	1.84
70	*Nature Medicine*	71	0.21	1.85
71	*The American Journal of Pathology*	70	0.20	1.85
72	*Journal of Neurophysiology*	68	0.20	1.86
73	*Neurology*	68	0.20	1.86
74	*Ophthalmic Genetics*	64	0.18	1.87
75	*Seminars in Ophthalmology*	64	0.18	1.88
76	*Journal of Cell Biology*	63	0.18	1.88
77	*Biomedical Optics Express*	62	0.18	1.89
78	*eLife*	62	0.18	1.89
79	*Human Mutation*	62	0.18	1.90
80	*Ophthalmic Epidemiology*	61	0.18	1.90
81	*Biochemical and Biophysical Research Communications*	59	0.17	1.91
82	*Journal of Lipid Research*	59	0.17	1.91
83	*Visual Neuroscience*	59	0.17	1.92
84	*Documenta Ophthalmologica*	58	0.17	1.92
85	*CDSR*	57	0.16	1.93
86	*Journal of Clinical Medicine*	56	0.16	1.93
87	*Development*	55	0.16	1.94
88	*Nucleic Acids Research*	55	0.16	1.94
89	*Cell Reports*	53	0.15	1.95
90	*Journal of Experimental Psychology*	53	0.15	1.95
91	*Journal of Ocular Pharmacology and Therapeutics*	52	0.15	1.96
92	*Frontiers in Immunology*	51	0.15	1.96
93	*FASEB*	50	0.14	1.97
94	*Ophthalmic Research*	50	0.14	1.97
95	*Clinical and Experimental Optometry*	49	0.14	1.98
96	*American Journal of Medical Genetics*	47	0.14	1.98
97	*Lancet Global Health*	47	0.14	1.99
98	*Biochemistry*	46	0.13	1.99
99	*Journal of Immunology*	45	0.13	2.00
100	*Klin Monatsbl Augenheilkd*	45	0.13	2.00
101	*Contact Lens and Anterior Eye*	42	0.12	2.00
102	*Journal of Clinical Oncology*	42	0.12	2.01
103	*Journal of General Physiology*	42	0.12	2.01
104	*Journal of Medical Genetics*	42	0.12	2.02
105	*Asia-Pacific Journal of Ophthalmology*	41	0.12	2.02
106	*Diabetologia*	41	0.12	2.03
107	*IEEE*	41	0.12	2.03
108	*Annual Review of Vision Science*	40	0.12	2.03
109	*BioMed Research International*	40	0.12	2.04
110	*BMJ Open*	40	0.12	2.04
111	*Brain*	40	0.12	2.05
112	*Cancer Research*	40	0.12	2.05
113	*Eye and Vision*	40	0.12	2.05
114	*PLOS Genetics*	40	0.12	2.06
115	*International Ophthalmology Clinics*	39	0.11	2.06
116	*American Journal of Ophthalmology Case Reports*	38	0.11	2.06

(*continued on next page*)

Supplementary Table 1. (continued)

Rank	Journal	Number of Citations	% of Total Citations	Log-Rank
117	*Nature Reviews Molecular Cell Biology*	38	0.11	2.07
118	*Arquivos Brasileiros de Oftalmologia*	37	0.11	2.07
119	*Brain Research*	37	0.11	2.08
120	*Journal of Cell Science*	37	0.11	2.08
121	*Journal of Clinical Endocrinology & Metabolism*	37	0.11	2.08
122	*Journal of Pediatric Ophthalmology & Strabismus*	37	0.11	2.09
123	*Molecular Therapy*	37	0.11	2.09
124	*Biophysical Journal*	36	0.10	2.09
125	*Journal of Neurochemistry*	36	0.10	2.10
126	*Melanoma Research*	36	0.10	2.10
127	*Cell Death & Disease*	35	0.10	2.10
128	*Pediatrics/AAP*	35	0.10	2.11
129	*Journal of Ophthalmic and Vision Research*	34	0.10	2.11
130	*Ophthalmologe*	34	0.10	2.11
131	*Genetics in Medicine*	33	0.10	2.12
132	*Korean Journal of Ophthalmology*	33	0.10	2.12
133	*Stem Cells*	33	0.10	2.12
134	*Clinical Cancer Research*	32	0.09	2.13
135	*Nature Reviews Neuroscience*	32	0.09	2.13
136	*Retinal Cases and Brief Reports*	32	0.09	2.13
137	*Cellular and Molecular Life Sciences*	31	0.09	2.14
138	*Cells*	31	0.09	2.14
139	*Journal of Cellular Physiology*	31	0.09	2.14
140	*Ophthalmic Plastic & Reconstructive Surgery*	31	0.09	2.15
141	*Cognition*	30	0.09	2.15
142	*Human Genetics*	30	0.09	2.15
143	*Journal of Cellular and Molecular Medicine*	30	0.09	2.16
144	*Alzheimer's & Dementia Journal*	29	0.08	2.16
145	*Autophagy*	29	0.08	2.16
146	*The EMBO Journal*	29	0.08	2.16
147	*FEBS Letters*	29	0.08	2.17
148	*Medicine*	29	0.08	2.17
149	*Nature Biotechnology*	29	0.08	2.17
150	*Cell Death & Differentiation*	28	0.08	2.18
151	*Developmental Biology*	28	0.08	2.18
152	*European Journal of Human Genetics*	28	0.08	2.18
153	*Frontiers in Cellular Neuroscience*	28	0.08	2.18
154	*Journal Francias d'Ophthalmologie*	28	0.08	2.19
155	*Molecular Cell*	28	0.08	2.19
156	*Bioinformatics*	27	0.08	2.19
157	*BMJ Open Ophthalmmology*	27	0.08	2.20
158	*Frontiers in Neuroscience*	27	0.08	2.20
159	*Journal of Neurology, Neurosurgery, and Psychiatry*	27	0.08	2.20
160	*American Journal of Physiology-Cell Physiology*	26	0.08	2.20
161	*Biochimica et Biophysica Acta*	26	0.08	2.21
162	*Developmental Cell*	26	0.08	2.21
163	*European Journal of Neuroscience*	26	0.08	2.21
164	*Genes*	26	0.08	2.21
165	*Ophthalmology Glaucoma*	26	0.08	2.22
166	*Ophthalmology and Therapy*	26	0.08	2.22
167	*Physiological Reviews*	26	0.08	2.22
168	*Biochemical Journal*	25	0.07	2.23
169	*Blood*	25	0.07	2.23
170	*Perception*	25	0.07	2.23
171	*Cancers*	24	0.07	2.23
172	*Circulation Research*	24	0.07	2.24
173	*Frontiers in Neurology*	24	0.07	2.24
174	*Journal of Neurology*	24	0.07	2.24
175	*Perception & Psychophysics*	24	0.07	2.24
176	*PLOS Computational Biology*	24	0.07	2.25

(*continued on next page*)

ARTICLE

Supplementary Table 1. (continued)

Rank	Journal	Number of Citations	% of Total Citations	Log-Rank
177	*Accident Analysis & Prevention*	23	0.07	2.25
178	*BMJ*	23	0.07	2.25
179	*Frontiers in Cell and Developmental Biology*	23	0.07	2.25
180	*International Journal of Epidemiology*	23	0.07	2.26
181	*Journal of Alzheimer's Disease*	23	0.07	2.26
182	*Nature Cell Biology*	23	0.07	2.26
183	*Nature Reviews Genetics*	23	0.07	2.26
184	*Ocular Oncology and Pathology*	23	0.07	2.26
185	*Psychological Science*	23	0.07	2.27
186	*Redox Biology*	23	0.07	2.27
187	*Trends in Cognitive Sciences*	23 (25,188*)	0.07 (72.73*)	2.26

[a]Denotes accumulation.

*Accumulation of citations.
